# Therapeutic Potential of an Endolysin Derived from Kayvirus S25-3 for Staphylococcal Impetigo

**DOI:** 10.3390/v11090769

**Published:** 2019-08-22

**Authors:** Ichiro Imanishi, Jumpei Uchiyama, Toshihiro Tsukui, Junzo Hisatsune, Kaori Ide, Shigenobu Matsuzaki, Motoyuki Sugai, Koji Nishifuji

**Affiliations:** 1 Laboratory of Veterinary Internal Medicine, Division of Animal Life Science, Institute of Agriculture, Graduate School, Tokyo University of Agriculture and Technology, 3-5-8 Saiwai-cho, Fuchu, Tokyo 183-8509, Japan; 2 Laboratory of Veterinary Microbiology I, School of Veterinary Medicine, Azabu University, 1-17-71 Fuchinobe, Chuo-ku, Sagamihara, Kanagawa 252-5201, Japan; 3 Nippon Zenyaku Kogyo Co. Ltd., 1-1 Tairanoue, Sasagawa, Asaka-machi, Koriyama, Fukushima 963-0196, Japan; 4 Department of Bacteriology, Graduate school of Biomedical and Health Sciences, Hiroshima University, 1-2-3 Kasumi, Minami-ku, Hiroshima 734-8553, Japan; 5 Department of Ophthalmology and Visual Science, Kochi Medical School, Kochi University, 185-1 Kohasu, Oko-cho, Nankoku, Kochi 783-8505, Japan

**Keywords:** *Staphylococcus aureus*, bacteriophage, endolysin, kayvirus, cutaneous microbiome, impetigo

## Abstract

Impetigo is a contagious skin infection predominantly caused by *Staphylococcus aureus*. Decontamination of *S. aureus* from the skin is becoming more difficult because of the emergence of antibiotic-resistant strains. Bacteriophage endolysins are less likely to invoke resistance and can eliminate the target bacteria without disturbance of the normal microflora. In this study, we investigated the therapeutic potential of a recombinant endolysin derived from kayvirus S25-3 against staphylococcal impetigo in an experimental setting. First, the recombinant S25-3 endolysin required an incubation period of over 15 minutes to exhibit efficient bactericidal effects against *S. aureus*. Second, topical application of the recombinant S25-3 endolysin decreased the number of intraepidermal staphylococci and the size of pustules in an experimental mouse model of impetigo. Third, treatment with the recombinant S25-3 endolysin increased the diversity of the skin microbiota in the same mice. Finally, we revealed the genus-specific bacteriolytic effect of recombinant S25-3 endolysin against staphylococci, particularly *S. aureus*, among human skin commensal bacteria. Therefore, topical treatment with recombinant S25-3 endolysin can be a promising disease management procedure for staphylococcal impetigo by efficient bacteriolysis of *S. aureus* while improving the cutaneous bacterial microflora.

## 1. Introduction

Impetigo, a bacterial skin infection, is highly contagious among patients and mainly affects children [1]. Over 162 million children have been reported to have suffered from impetigo [2]. Impetigo is generally categorized into two clinical phenotypes, bullous and non-bullous impetigo, and *Staphylococcus aureus* is a major pathogen in both phenotypes [3]. After the pustules rupture, *S. aureus* can be transmitted to other body sites or other people [3,4]. As a result of the infection process, outbreaks of impetigo are reported in health care facilities and nursery schools [5]. Thus, effective treatment and prevention of bacterial transmission are crucial for the management of impetigo.

In patients with impetigo, topical or systemic antibiotics are recommended as the first-line choice for treatment [3]. Systemic antibiotics are frequently used when lesions become larger, and topical medications become impractical [3,6]. However, the frequent use of antibiotics has led to the emergence of new drug-resistant bacteria [6,7], which has recently been considered a major threat to public health [1,6]. Antibiotic therapy also disturbs the normal skin microflora [8]. Such medication eliminates other useful commensal bacteria that protect the skin from virulent bacteria [6,8]. Thus, new interventions that are less prone to elicit resistance and specifically kill virulent *S. aureus* are needed as an alternative to antibiotics.

Bacteriophage (phage) therapy has received attention as an alternative method to treat drug-resistant bacteria [9,10]. Instead of the phage itself, the use of endolysin (a phage-encoded bacteriolytic enzyme that degrades bacterial peptidoglycans) has been attempted to provide a therapeutic agent for various bacterial infections [11,12]. Because of the narrow target spectrum of endolysin, it is considered to eliminate specific bacteria without disturbing the normal bacterial microflora [10,13,14]. Several studies have reported that the development of endolysin resistance is less likely than the development of antibiotic resistance [9,10,15]. The administration of endolysin has been shown to be effective against bacteremia and pneumonia caused by *S. aureus* in mice [15,16]. Because of its effectiveness against *S. aureus*, an endolysin cream has been authorized as a class 1 medical device in Europe [17]. However, the efficacy of endolysin is not always adequate for the treatment of bacterial infections in humans [18]. Thus, the efficacy of phage therapy should be thoroughly and carefully studied before clinical use.

Among the endolysins isolated from *Staphylococcus* phages, those derived from the family *Herelleviridae* genus kayvirus are the most well studied [9,10]. The endolysin protein sequences are highly conserved among different viral strains belonging to the kayvirus genus [10,19]. The kayvirus endolysins show bacteriolytic and bactericidal activity against *Staphylococcus* spp. including *S. aureus* [10,13,14]. Although the clinical examination of patients with skin infections, atopic dermatitis, and bacteremia that have been treated with endolysin as an anti-staphylococcal therapy have been conducted [20,21], the efficacy against impetigo, a common skin problem in humans, has not been sufficiently investigated. To apply kayvirus endolysins for the management of impetigo, investigations into the compatibility of endolysin to the management of impetigo in an experimental setting are required.

We previously reported another kayvirus, designated S25-3 [22,23]. In this study, we investigated whether an endolysin derived from kayvirus S25-3 could be used to manage experimental staphylococcal impetigo.

## 2. Materials and Methods

### 2.1. Production and Purification of Recombinant Protein

Phage genomic DNA was obtained as previously described [22]. Based on the available genetic information for kayvirus S25-3 (GenBank accession No.: YP_008854201), primer sets for the S25-3 endolysin gene were designed. After amplification of the genomic DNA by PCR with the primer set (5’-CCCCGGTACCATGGCTAAGACTCAAGCAGAAATAAATAAACG-3’ and 5’-CCCCAAGCTTCTAATGATGATGATGATGATGCTTGAATACTCCCCAAGCAACACCA-3’), the DNA fragment with the target sequence was subcloned into the pCold III vector (Takara Bio, Shiga, Japan), resulting in plasmid pCold III S25-3LYS. Plasmid pCold III was used as a negative control. Plasmids pCold III S25-3LYS and pCold III were transformed into *Escherichia coli* NiCo21 (DE3) (New England BioLabs, Ipswich, MA, USA) and the proteins were overexpressed in accordance with the manufacturer’s instructions. *E. coli* NiCo21 (DE3) clones were cultured in Luria–Bertani broth (LB broth, Miller; Nacalai Tesque, Kyoto, Japan) supplemented with 100 μg/mL ampicillin.

Bacterial cells were sonicated in a sonication solution (100 mM sodium phosphate, 300 mM NaCl, pH 7.8) and the cell lysate was incubated with Co2+-agarose resin (ProteNova, Kagawa, Japan) overnight at 4 °C. After the resin was washed with the sonication solution, the proteins were eluted with elution buffer (50 mM sodium phosphate, 300 mM NaCl, pH 7.8 supplemented with 5 mM or 350 mM imidazole). The eluates with 350 mM imidazole contained the recombinant proteins. The eluates with 350 mM imidazole derived from NiCo21 (DE3) [pCold III S25-3LYS], and the corresponding fractions derived from NiCo21 (DE3) [pCold III], were dialyzed against phosphate-buffered saline (PBS; pH 7.4). After separation of the proteins by SDS-PAGE and staining with Coomassie G-250 solution (Life Technologies, Carlsbad, CA, USA), the protein integrity was confirmed. The protein concentration of S25-3LYS-his was measured by a BCA kit (Thermo Fisher Scientific, Waltham, MA, USA). After dialysis, the fraction obtained from NiCo21 (DE3) [pCold III], corresponding to the fraction containing S25-3LYS-his, was used as a negative control solution.

### 2.2. Bacterial Strains

*S. aureus* strains COL [24], N315 [25], TY767, TF3030 [26], TY825 [27], N1 [28], and N2 [28] were used to examine the bacteriolytic and bactericidal activities of S25-3LYS-his. *S. aureus* strain TY825, which is a methicillin-resistant *S. aureus* strain producing exfoliative toxin B, a virulent factor of bullous impetigo [27], was used for topical inoculation of the mouse pinnae.

*Staphylococcus epidermidis* strains ATCC12228 [24], W860371, and M890190, *Streptococcus mitis* strain GTC495 [29], and *Pseudomonas aeruginosa* strains MS5639, MS5640, MS5641, PAO1 [30], D4 [30], S10 [30], and PA29 [30] were used to measure the lytic spectrum of the endolysin. The human commensal skin bacteria were also isolated as described below (see Table S2 in the supplemental material).

### 2.3. Bacterial Isolation from Human Skin

Twelve human volunteers, including four females and eight males, between 24 and 49 years old, were included in this study. Skin swabs were taken from three anatomic sites, including the forehead, the flexor part of the left forearm, and the dorsal skin. At each site, a 1 × 1-cm square of the skin surface was swabbed 30 times using a sterile cotton swab with constant pressure. These volunteers were informed of the study’s goals and provided written consent for participation before they were included in the study. All experiments using human resources were approved by the Ethical Committee at the Tokyo University of Agriculture and Technology (#30-04).

The bacteria were isolated from the swabs by growth on three culture media: tryptic soy agar (Beckton Dickinson and Co. Franklin Lakes, NJ, USA), Brucella HK medium (Kyokuto Pharmaceutical Industrial, Tokyo, Japan), and Hoyle’s tellurite agar (Beckton Dickinson and Co.). Bacteria were cultured on tryptic soy agar at 35 °C for 3 days under aerobic conditions, on Brucella HK medium at 37 °C for 3 days under anaerobic conditions, and on Hoyle’s tellurite agar at 37 °C for 5 days under aerobic conditions. Purification was performed at least three times. After the purification procedure, the bacterial cells were collected from the agar plates for phylogenetic classification and bacteriolytic assessment.

### 2.4. Phylogenetic Classification of Bacteria

After isolating the bacterial DNA, bacterial 16S rDNA fragments were amplified by PCR using a Bacterial 16S rDNA PCR Kit (Takara Bio). PCR products were sequenced with the BigDye Terminator v3.1 cycle sequencing kit (Life Technologies) using a Model 3130 Genetic Analyzer (Life Technologies), according to the manufacturer’s instructions. Sequences were phylogenetically assessed using the Ribosomal Database Project (RDP) (https://rdp.cme.msu.edu/) [31] and BLASTp at the National Center for Biotechnology Information (https://blast.ncbi.nlm.nih.gov/Blast.cgi).

### 2.5. Measurement of the Bacteriolytic and Bactericidal Activities

The bacteriolytic activity of S25-3LYS-his was assessed. Bacteria were cultured on appropriate media (see Table S2 in the supplemental material). Bacterial cells were washed and resuspended in sterile PBS containing 5 mM NaCl. The optical density was adjusted to ~1.0 at 600 nm using a spectrophotometer (Bio Spectrometer; Eppendorf, Hamburg, Germany). Then, 195 μL of *S. aureus* suspension was supplemented with 5 μL of S25-3LYS-his (400 μg/mL) or control solution as a negative control in a flat-bottomed 96-well microplate (Life Technologies). Preliminary studies revealed that the above concentrations of S25-3LYS-his and *S. aureus* suspension yielded the most effective bacteriolytic activity against *S. aureus*. The microplate was incubated for an appropriate period at 37 °C, and the optical density at 595 nm was measured over time using an iMark plate reader (BioRad Laboratories, Hercules, CA, USA). The reaction rate (∆OD_595_/min/mg) was calculated using a formula described in Figure S2 in the supplemental material.

The bactericidal activity of S25-3LYS-his was also assessed. Bacteria were cultured to mid-log phase, then 10 µL of endolysin (1 mg/mL) or control solution was added to 190 μL of bacterial suspension (1.6 × 10^8^ CFU/mL). After incubation at 37 °C for the appropriate period, the bacterial suspension was plated on mannitol salt agar (Nissui Pharmaceuticals, Tokyo, Japan). After incubation (37 °C for 48 h), the viable bacteria were counted.

The rate of turbidity reduction (ΔOD_595_), bacterial reduction (ΔCFU/mL), and the bacteriolytic reaction rate within 15 min (ΔOD_595_/min/mg) were calculated using a formula described in Figure S2 in the supplemental material.

### 2.6. Application of S25-3LYS in a Mouse Model of Impetigo

*S. aureus* strain TY825 was cultured in LB broth (LB Broth, Miller; Nacalai Tesque) until mid-log-phase, and was then washed with PBS three times. Six-week-old, female, BALB/c Cr Slc mice (Sankyo Labo Service, Tokyo, Japan) were used. After tape-stripping was performed on the murine inner pinnae seven times, a 1 × 1-cm gauze containing 2.0 × 10^8^ CFU of *S. aureus* was adhered to the right and left pinnae. Two microliters of S25-3LYS-his (1 mg/mL) and the same volume of control solution were applied to the gauze attached to the right and left pinnae, respectively. Six hours after inoculation, the mice were euthanized by cervical dislocation, and the pinnae were collected for further experiments.

The animal experiment was approved by the Animal Research Committee at Tokyo University of Agriculture and Technology (#27-40) and was performed under the International Guiding Principles for Biomedical Research Involving Animals.

### 2.7. Enumeration of Bacterial Densities in the Mouse Pinnae

Mouse pinnae were suspended and mixed in 500 µL of PBS, and the suspension was plated onto mannitol salt agar. After incubation (37 °C for 48 h), the appearance of bacterial colonies was enumerated.

### 2.8. Histopathological and Immunofluorescence Analyses

The mouse pinnae tissues were fixed with 10% formaldehyde (*N* = 6, each group). Each tissue was dissected into 10 pieces and embedded in paraffin. After paraffin-embedded samples were serially sectioned, the sections were deparaffinized. For histopathological analysis, tissue sections were stained with hematoxylin and eosin and then observed.

For immunofluorescence analysis, the deparaffinized sections were incubated with primary and then secondary antibodies. As primary antibodies, mouse anti-human keratin type 1 and 2 monoclonal antibody (clone AE1+AE3; Progen Biotechnik GmbH; Heidelberg, Germany) and rabbit anti-*Staphylococcus* polyclonal antibody (clone CH91; courtesy of Makoto Haritani, National Institute of Animal Health, Ibaraki, Japan) [32] were used. As secondary antibodies, Alexa Fluor™ 546-conjugated goat anti-rabbit IgG and Alexa Fluor™ 488 goat anti-mouse IgG (Life Technologies) were used. Nuclei were counterstained with Hoechst 33258 (Life Technologies).

Tissue sections were observed using a light and fluorescence microscope (BX43 F; Olympus, Tokyo, Japan). Micrographic images were recorded using image software (CellSens Standard; Olympus). The length of the basement membrane (BM), the dimensions and number of intra-epidermal clefts, and the number of intra-epidermal staphylococci, were quantified by previously described methods [28].

### 2.9. Microbiota Analysis

The pinnae tissues were suspended in 500 μL of sterile PBS and mixed. After dilution, 100 μL of the pinna solution was plated onto tryptic soy agar and incubated at 37 °C for 48 h under aerobic conditions. Ten colonies per pinna were randomly isolated. Phylogenetic classification of the bacteria was performed by sequencing the 16S rRNA gene as described above.

### 2.10. Microbiome Analysis

The DNA was purified directly from tissue samples obtained from the mouse pinnae using an ISOSPIN Fecal DNA kit (Nippon Gene, Toyama, Japan), in accordance with the manufacturer’s instructions. The V3–V4 region of the 16S rRNA gene [33] was amplified from 500 ng of extracted DNA using the optimized methods for Illumina MiSeq (Illumina, San Diego, CA, USA) and barcoded sample-specific primers (forward, 5’–TCGTCGGCAGCGTCAGATGTGTATAAGAGACAGCCTACGGGNGGCWGCAG–3’; reverse, 5’–GTCTCGTGGGCTCGGAGATGTGTATAAGAGACAGGACTACHVGGGTATCTAATCC–3’). Each PCR product was purified using an Agencourt AMPure XP Beads Kit (Beckman Coulter, Pasadena, CA, USA) and quantified using a Qubit dsDNA BR Assay Kit (Thermo Fisher Scientific). Each 100-ng amplicon underwent a second round of PCR for indexing, using a Nextera XT Index Kit v2 (Illumina). After purification, the PCR products were quantified by NanoPhotometer (Implen, Westlake Village, CA, USA) and pooled into one tube with a final concentration of 1.6 ng/μL. The concentration of the pooled DNA library was validated using an Agilent 2100 Bioanalyzer (Agilent, Santa Clara, CA, USA). After denaturation with NaOH, 850 μL of a 9 pM DNA library and 150 μL of 9 pM PhiX were mixed and subjected to pair-end sequencing using Illumina MiSeq with a MiSeq Reagent Kit v3 (Illumina).

The sequence data were analyzed using Quantitative Insights into Microbial Ecology 2 (QIIME2) v2019.1.0 [34]. The DADA2 software package v2018.4.0 incorporated in QIIME 2 was used to correct amplicon sequence errors and to construct an amplicon sequence variant table [35]. The Greengenes 99% reference database v13.8 was used for the taxonomic classification of each amplicon sequence variant. The microbiome diversity was analyzed using a rarefied amplicon sequence variant table.

### 2.11. Statistical Analysis

Statistical analysis was performed using GraphPad Prism 6 (GraphPad Software Inc., San Diego, CA, USA). Comparisons of bacterial turbidity and bacterial reduction between control and S25-3LYS-his groups were performed using the Mann–Whitney U test. Correlations between turbidity reduction and bacterial reduction in S25-3LYS-his-treated groups were analyzed by Spearman’s correlation coefficient, and the least square regression line was calculated. The density of staphylococcal cells, number of staphylococcal cells in intra-epidermal tissue, and area and frequency of pustules in mouse skins were analyzed using the Mann–Whitney U test. The taxonomic abundance of viable bacteria from murine pinnae and the frequency of bacteria showing “sensitivity” in commensal bacteria on human skins were analyzed using the Chi-square test. Reaction rate of “sensitive” staphylococci by S25-3LYS-his were analyzed using the Kruskal–Wallis test.

Metrics of alpha diversity (i.e., Faith-Phylogenetic Diversity (PD), Chao1, and Shannon) and beta diversity (i.e., weighted and unweighted UniFrac) were examined using QIIME 2 [34]. The correlations of the metrics of alpha and beta diversities with age were statistically analyzed by Kruskal–Wallis and PERMANOVA tests, respectively, implemented in QIIME 2 [34].

## 3. Results and Discussion

### 3.1. Characteristics of the Endolysin Derived from Kayvirus S25-3

Amino acid sequence analysis revealed that the endolysin derived from kayvirus S25-3 shared 99.39% identity with the endolysin derived from kayvirus K, the first-isolated kayvirus endolysin [36]. The endolysin gene of kayvirus S25-3 was subcloned into a protein expression vector and transferred into *E. coli* to generate a recombinant endolysin fused with a 6× histidine tag at the C-terminus. This recombinant endolysin is termed S25-3LYS-his in this study. First, mass spectrometry analysis revealed that the cleavage sites of the *S. aureus* peptidoglycan by S25-3LYS-his were identical to those digested by endolysins of other kayviruses K, GH15, and SAP-1 (see Figure S1 in the supplemental material) [10,37,38]. Second, in vitro incubation of S25-3LYS-his with nine clinical isolates of *S. aureus* revealed that the reduction of bacterial turbidity and the bactericidal effects of S25-3LYS-his were observed within 15 min of incubation (see Table S1 in the supplemental material), which was similar to previous reports of kayvirus endolysins [11,37,38]. The purified S25-3LYS-his was used for the following studies. The purified solution prepared from *E. coli* harboring the protein expression vector without inserted DNA was used as a negative control.

### 3.2. Prolonged Incubation of S25-3 Endolysin with S. aureus Increases the Bactericidal Effect

Because endolysins have been considered to be rapid-acting antimicrobial agents [11,15,37,38], most of the previous studies examined the bacteriolytic and bactericidal effects of endolysins over a short time period. However, to apply endolysins as topical pharmaceutical agents for controlling bacterial skin infections, it is important to understand the appropriate exposure period of endolysins to obtain maximum bactericidal effects. Thus, we compared the bacteriolytic and bactericidal effects of S25-3LYS-his against *S. aureus* following application over short and long time periods.

**Figure 1:**
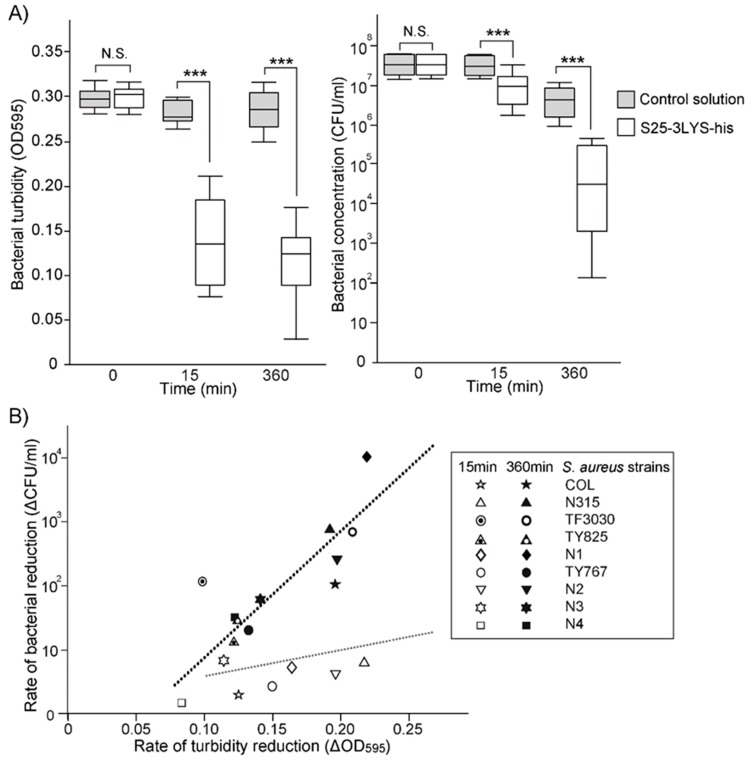
Description of S25-3LYS-his activities against *S. aureus*. (**A**) Comparison of bacterial turbidity (left) and bacterial reduction (right) between the control and S25-3LYS-his groups. At 15 min of incubation, bacterial turbidities (OD_595_) of the control and S25-3LYS-his groups were 0.284 ± 0.013 and 0.141 ± 0.005, respectively (mean ± SD). At 360 min of incubation, the bacterial turbidities of the control and S25-3LYS-his groups were 0.286 ± 0.023 and 0.115 ± 0.045, respectively. At 15 min of incubation, the bacterial concentrations (log_10_ CFU/mL) of the control and S25-3LYS-his groups were 7.41 ± 0.24 and 6.80 ± 0.39, respectively. At 360 min of incubation, the bacterial concentrations of the control and S25-3LYS-his groups were 7.41 ± 0.24 and 6.80 ± 0.39, respectively. Statistical significance is indicated as “***” (*P* < 0.005); no statistical significance is indicated as “N.S.”. (**B**) Correlation analysis between turbidity reduction and bacterial reduction in the S25-3LYS-his-treated groups, measured at 15 and 360 min. The regression lines are separately plotted around the data points at 15 min (*R^2^* = 0.22) and 360 min (*R^2^* = 0.76), which are shown in gray and black, respectively. The *S. aureus* strains tested are shown in the right-hand box.

First, we measured the changes in bacterial turbidity and the number of viable bacteria after 15 min and 360 min of incubation with S25-3LYS-his (Figure 1A). The reduction rate of bacterial turbidity and the number of viable bacteria (ΔOD_595_ and ΔCFU/mL) following S25-3LYS-his treatment were calculated using the formula described in Figure S2A and S2B in the supplemental materials, respectively. The correlations between the reduction rates of bacterial turbidity and the numbers of viable bacteria at 15 min and 360 min were also analyzed.

Our results revealed that the rate of turbidity reduction was not statistically correlated with the reduction of viable bacteria after 15 min of incubation (*P* > 0.05; Figure 1B). However, this correlation was observed after 360 min of incubation (*P* < 0.05; Figure 1B). This result suggested that a shorter exposure to S25-3LYS-his exhibited insufficient bactericidal activity against *S. aureus* strains, but efficient bacteriolytic activity. It is assumed that some populations of *S. aureus* become protoplasts following short exposure to S25-3LYS-his, as observed with other peptidoglycan-degrading enzymes such as lysozyme [39].

As bacteriolytic–bactericidal correlations can be represented as a regression line, we compared the bacteriolytic-bactericidal regression lines obtained after 15 min and 360 min of incubation. A higher slope of the regression line was associated with stronger bactericidal effects during constant bacteriolytic activity. The slope of the regression line at 360 min of incubation was 20.31, while that at 15 min of incubation was 4.43 (Figure 1B). These findings indicated that prolonged incubation with S25-3LYS-his increased the bactericidal effect against *S. aureus*.

### 3.3. Topical Application of S25-3 Endolysin Inhibits Staphylococcal Epidermal Invasion in Experimental Staphylococcal Impetigo

**Figure 2:**
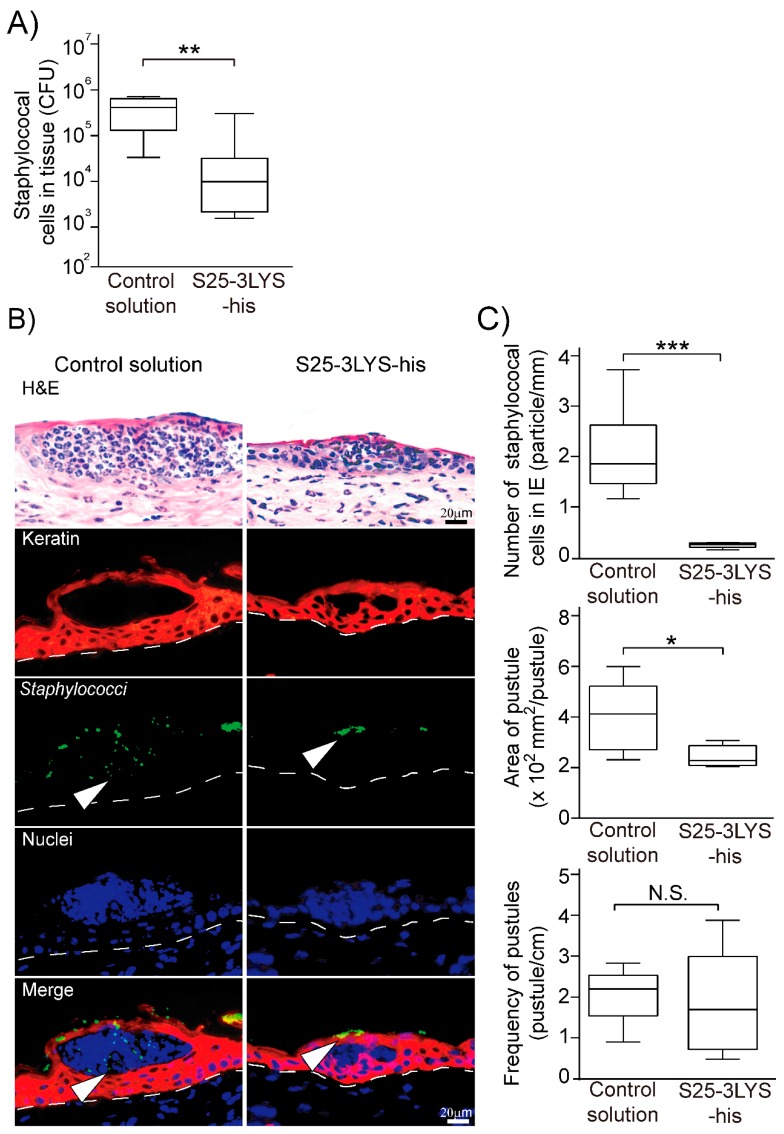
Evaluation of S25-3LYS-his treatment in an impetigo mouse model. (**A**) The density of staphylococcal cells in mouse skin. The densities of viable *Staphylococcus* spp. were measured using mannitol salt agar. The statistical significance is indicated as “**” (*P* < 0.01). Of note, when the total bacteria in the mouse skin was measured using tryptic soy agar, there was no statistical difference between the S25-3LYS-his-treated and control groups (*P* > 0.05; see Figure S4 in the supplemental material). (**B**) Pathological analysis of mouse skin tissues in the control solution (left) and S25-3LYS-his-treated solution (right). Representative photographs are shown. In the top micrographs, the tissues stained with hematoxylin and eosin (H&E) are shown. In the second from the top to the bottom micrographs, keratin, staphylococci, and nuclei-specific staining are shown in red, green, and blue, respectively. Staphylococci are indicated by white arrowheads (i.e., images on the third and the fifth rows from the top). The dotted lines indicate the basement membranes. (**C**) Staphylococcal cells in the intra-epidermal (IE) region, the area of pustules, and the frequency of pustules were measured by pathological analysis. Statistical significance is shown on the graph (*, *P* < 0.05; ***, *P* < 0.005); no statistical significance is indicated as “N.S.”

We previously generated a mouse model of staphylococcal impetigo by topical inoculation of *S. aureus* strains onto tape-stripped mouse skin [28]. In the mouse model, staphylococci adhere to the viable epidermis and recruit neutrophils, then penetrate into the epidermis through inter-keratinocyte clefts created by the neutrophils. In this study, we investigated the therapeutic effect of S25-3LYS-his in this experimental mouse model of impetigo. The S25-3LYS-his was applied onto tape-stripped murine pinnae after inoculation of the same area with *S. aureus*. At 6 h after inoculation, murine pinnae were collected, and the densities of staphylococci between the S25-3LYS-his-treated group and the control group were compared. The S25-3LYS-his-treated group showed a lower concentration of staphylococci than the control group (mean ± standard deviation, 4.69 ± 0.69 log_10_ CFU/tissue in S25-3LYS-his-treated group and 5.44 ± 0.46 log_10_ CFU/tissue in control group, *P* < 0.01; Figure 2A).

The tissues were also subjected to immunofluorescence analysis. Staphylococci were observed in intraepidermal clefts in the control group but were only detected on the skin surface in the S25-3LYS-his-treated group. The numbers of intraepidermal staphylococci per 1 mm of the BM in the S25-3LYS-his-treated group were lower than those in the control group (0.24 ± 0.06 bacteria/mm of BM in S25-3LYS-his-treated group and 2.07 ± 0.90 bacteria/mm of BM in control group, *P* < 0.005; Figure 2C). These results indicated that topical application of S25-3LYS-his inhibited *S. aureus* invasion of the epidermis in the experimental mouse model of impetigo.

The area of intraepidermal pustules and the frequency of pustules per 1 cm of BM were also analyzed in skin sections. The area of pustules in the S25-3LYS-his-treated group was smaller than in the control group (232.6 ± 40.9 μm^2^ in S25-3LYS-his-treated group and 391.7 ± 132.0 μm^2^ in control group, *P* < 0.05; Figure 2C), whereas the frequency of pustules was not statistically different between the two groups (1.90 ± 1.26/cm BM in S25-3LYS-his-treated group and 2.08 ± 0.64/cm BM in control group, *P* > 0.05; Figure 2C). These results indicated that S25-3LYS-his suppressed the enlargement of pustules in the mouse model of impetigo, although it did not inhibit pustule formation.

### 3.4. S25-3LYS-his Treatment Increases Bacterial Diversity in Mouse Skin

**Figure 3:**
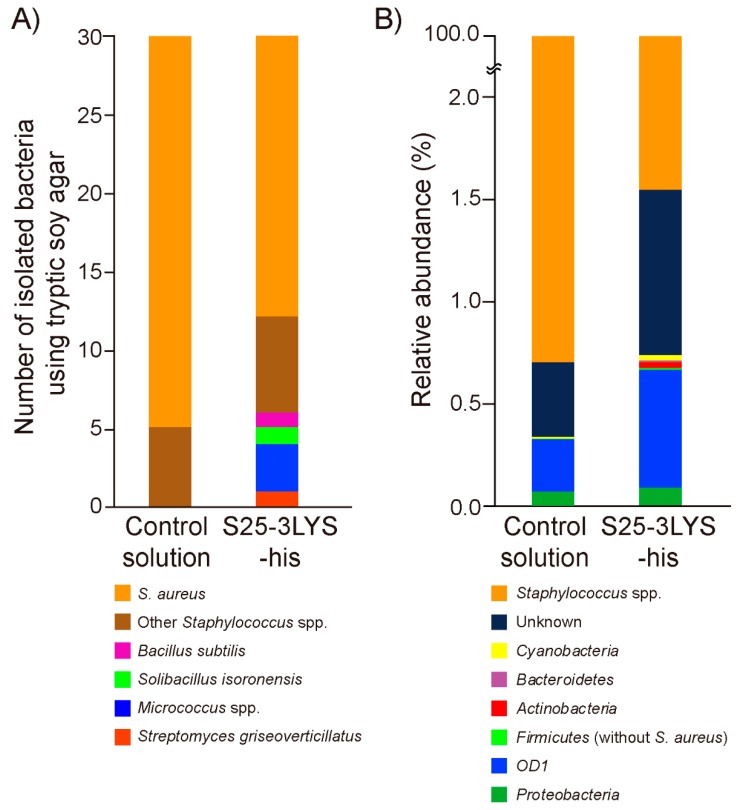
Taxonomical abundance of the microbiome on mouse skin treated with the control solution or S25-3LYS-his, as determined by (**A**) culture methods and (**B**) 16S rRNA gene amplicon analysis. At the bottom, bacterial taxonomical assignments are shown. Using the culture method, 30 bacteria were isolated from the skin (*N* = 3) for each treatment group, then phylogenetically classified based on their 16S rRNA gene sequence. Using 16S rRNA gene amplicon analysis, the average taxonomical abundance of the skin microbiome (*N* = 3) for each treatment group is shown. The phylum taxonomical level is shown. For data analysis, a total of 182,317 reads were used, with each sample comprising 30,386.2 ± 5886.1 reads (mean ± SD), of which 27,700 reads in each sample were rarefied.

Because kayvirus endolysins are thought to lyse *Staphylococcus* spp. selectively [40], restoration of the microbial balance on skin infected by staphylococci may be achievable by the application of kayvirus endolysins. Therefore, the skin microbial diversities of the S25-3LYS-his-treated group and the control group were analyzed. We revealed that the taxonomic abundance of viable bacteria, which were harvested from the murine pinnae and cultured on tryptic soy agar, was significantly higher in the S25-3LYS-his treatment group than in the control group (*P* < 0.05; Figure 3A).

Taxonomic annotation using the bacterial 16S rRNA sequence database revealed that over 98% of bacteria in both the S25-3LYS-his-treated group and the control group were *Staphylococcus* spp. (Figure 3B). However, the analysis of alpha diversity revealed that Chao1 and phylogenic diversity (Faith-PD) indexes in the S25-3LYS-his-treated group were significantly higher than the control group (*P* < 0.05; see Figure S3 in the supplemental material), whereas there were no statistical differences in the Shannon index between the two groups (*P* = 0.5127). Considering that Chao1 and Faith-PD explain species richness, while Shannon index explains both richness and evenness of the species present, our findings indicate that S25-3LYS-his increased the alpha diversity of the bacterial microbiome on the skin surface, although *S. aureus* was still predominant in the S25-3LYS-his-treated group.

By contrast, the analysis of beta diversity revealed that there were no statistical differences in the weighted UniFrac and unweighted UniFrac values between the two groups (*P* = 0.7040 and 0.1840 in weighted UniFrac and unweighted UniFrac, respectively). At a minimum, these findings indicated that topical application of S25-3LYS-his increased bacterial richness. It is worth noting that 16S rRNA amplicon analysis could not discriminate between the sequences of dead bacteria and those of viable bacteria, or between *S. aureus* and other staphylococcal species.

### 3.5. S25-3 Endolysin Preferentially Lyses S. aureus among Human Skin Bacteria

We further examined the lytic activity of S25-3LYS-his against commensal bacteria on human skin in vitro. A collection of human skin commensal bacteria was prepared, which included 11 reference strains and 89 originally isolated bacteria from skin swabs of healthy individuals. It has been reported that three genera (*Staphylococcus*, *Corynebacterium*, and *Propionibacterium*) constitute over 60% of the bacterial species present in the human skin microbiota [41,42]. In addition to these three genera, other bacteria including *S. mitis*, *P. aeruginosa*, *Bacillus* spp., *Micrococcus* spp., and *Brevibacterium* spp. were also isolated in our study (see Figure S2 in the supplemental material).

**Figure 4:**
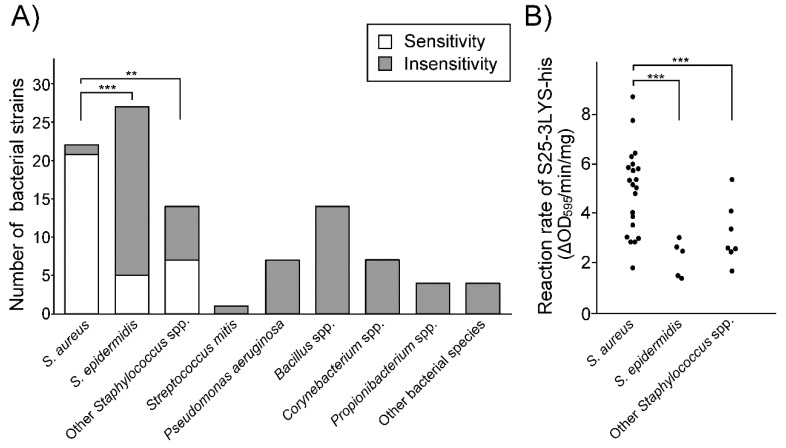
Lytic spectra of S25-3LYS-his on cutaneous bacteria. (**A**) Only staphylococci showed sensitivity to S25-3LYS-his. Strains showing “sensitivity” and “insensitivity” to S25-3LYS-his are shown in white and black squares, respectively. (**B**) Bacteriolytic reaction rates of S25-3LYS-his (ΔOD_595_/min/mg) to *Staphylococcus* spp. showing sensitivity to S25-3LYS-his. Statistical significance is shown on the graph (*, *P* < 0.05; **, *P* < 0.01; ***, *P* < 0.005).

Bacterial sensitivity was determined by comparing bacterial turbidity between the S25-3LYS-his-treated group and the control group after 360 min of incubation. In this experiment, “sensitivity” meant that bacterial turbidity in the S25-3LYS-his-treated group was significantly lower than in the control group. By contrast, “insensitivity” meant that there were no statistical differences in turbidity between the two groups. We found that the frequency of bacteria showing “sensitivity” was significantly higher in *S. aureus* strains than in *S. epidermidis* strains (*P* < 0.005) and other staphylococcal strains (*P* < 0.01; Figure 4A).

Moreover, the bacteriolytic reaction rate of “sensitive” staphylococci induced by S25-3LYS-his (ΔOD_595_/min/mg S25-3LYS-his; see Figure S2C in the supplementary material) was examined. The reaction rates were significantly higher in *S. aureus* strains (*N* = 21) than in *Staphylococcus epidermidis* strains (*N* = 5, *P* < 0.005) and other staphylococcal strains (*N* = 7, *P* < 0.005; Figure 4B). These findings indicated that S25-3LYS-his preferentially lysed *S. aureus* among human skin commensal bacteria.

### 3.6. Future Prospects for Endolysin Application to Treat Staphylococcal Skin Infections

In the present study, we showed that topical application of a recombinant endolysin derived from kayvirus S25-3 suppressed the enlargement of pustules and percutaneous invasion of staphylococci in an experimental model of impetigo. Further, the recombinant S25-3 endolysin preferentially decontaminated *S. aureus* with minimal effects on other skin commensal bacteria. Considering the similarities between S25-3 endolysin and other kayvirus endolysins in protein sequence and antimicrobial properties, our findings suggest that kayvirus endolysins have therapeutic potential for impetigo. To introduce kayvirus endolysin as a pharmaceutical agent for impetigo management successfully, further examination of the productization and safety of the endolysins should be considered.

For productization, the endolysin requires optimization as a pharmaceutical agent with good usability. First, because the topical application of endolysin over an extended period is expected to exhibit maximal bactericidal effects, an appropriate carrier material is needed for maintenance of endolysin on the skin. Second, considering the dissemination of the endolysin product, the endolysin needs to show decontamination effects superior to those of conventional agents. kayvirus endolysins are too large to penetrate the viable epidermis [43,44] and water-soluble proteins have difficulty penetrating the lipid bilayer in the stratum corneum; both of these factors present challenges in accessing *S. aureus* in the tissues. To effectively control the infection, the permeability of endolysin needs to be improved, potentially by developing a strategy of efficient transdermal drug delivery. Third, although endolysins are highly stable in general [45,46], the stability ofkayvirus endolysin combined with carrier materials therefore needs to be examined [47] to ensure that other components of the endolysin product do not disable its stability. Fourth, as some cases of non-bullous impetigo are caused by *Streptococcus pyogenes* and endolysins specific to *Streptococcus* spp. have been reported [48], the latter endolysins should be combined with kayvirus endolysins for the effective treatment of impetigo.

The safety of the long-term usage of topical endolysin products needs to be investigated. First, kayvirus endolysin is generally recognized as a safe agent because no adverse effects have been reported in pre-clinical and clinical trials [37,49,50]. In addition, a recent clinical trial revealed that systemic administration of an endolysin was effective for controlling *S. aureus* bacteremia in humans [49]. However, because the safety of the long-term usage of topical endolysin has not been confirmed, we believe that safety analysis of kayvirus endolysin is required. The repeated exposure to protein on the skin over a long period is thought to increase the risk of hypersensitivity [51]. The production of anti-endolysin antibodies has been shown as a result of topical application of chimeric endolysin in a murine model [52]. Some other lytic enzymes, such as egg lysozyme, have been shown to cause allergic reactions in both humans and dogs [53,54]. Endolysin treatment is not expected to lead to the appearance of resistant strains [13,15]. However, the long-term use of endolysin in clinical settings may lead to the emergence of endolysin-resistant bacteria.

Our findings suggest that the topical application of kayvirus endolysins to the site of infection is effective for controlling impetigo by decontamination of *S. aureus* while maintaining the robustness of the skin microbiome. In addition to applying kayvirus endolysin for the management of impetigo, endolysin can be applied to decontaminate virulent *S. aureus* from potential carriers. The endolysin cream can be prophylactically applied to individuals at risk of infection in certain settings, e.g., kindergarten or elementary school, where the hands of individuals are most likely to be contaminated with virulent *S. aureus* [55,56]. When kayvirus endolysin is topically applied to the hands after washing, it potentially prevents the spread of virulent *S. aureus* to the environment as a result of its efficient bacteriolytic activity and could therefore be used to prevent the spread of virulent *S. aureus* among children. With further study, endolysin can potentially be applied for the management of various staphylococcal skin infections in clinical settings.

## Supplementary Materials

The following are available online at https://www.mdpi.com/1999-4915/11/9/769/s1, Table S1: *S. aureus* strains used in this study, Table S2: Collection of human cutaneous bacteria used in this study, Figure S1: Analysis of *S. aureus* peptidoglycan reacted with S25-3LYS-his, using liquid chromatography coupled with quadruple time-of-flight mass spectrometry (QTOF), Figure S2: Mathematical formulas for (A) rate of turbidity reduction, (B) rate of bactericidal reduction, and (C) bacteriolytic reaction rate of S25-3LYS-his, Figure S3: Alpha diversity of mouse pinna microbiome, and Figure S4: Bacterial density of mouse pinnae, measured by culture method.
