# Supplementary material for: Therapeutic Potential of an Endolysin Derived from Kayvirus S25-3 for Staphylococcal Impetigo
Source: Viruses. 2019 Aug 22;11(9):769. doi: 10.3390/v11090769 (PMC6784202; doi:10.3390/v11090769)
Supplement: Supplementary file 1 [file viruses-11-00769-s001.pdf]

Table S1. *S. aureus* strains used in this study.

Strain	Origin	ST[a]	CC[b]	*mecA*[c]	*SCCmec*[d]	Virulence genes			Reaction rate	Reference
						et[c]	*pvl*[c]	*tsst*[c]	(Δ OD_{595}/mg/ml)	
COL	HA-MRSA reference strain	250	5	+	I	*etb*	-	+	2.678	[24]
N315	HA-MRSA reference strain	5	5	+	II	-	+	-	4.467	[25]
TF3030	Clinical isolation	30	30	+	IVc	*etb*	+	+	5.333	[26]
TY825	Clinical isolation	1442	89	+	IVc	*etb*	-	-	4.867	[27]
N1	Clinical isolation	4686	630	+	IVc	*etb*	-	+	2.889	[28]
TY767	Clinical isolation	121	121	+	V	*eta/etb*	-	-	8.356	This study
N2	Clinical isolation	1155	101	+	N.E.	*etb*	-	-	2.711	[28]
N3	Nasal cavity of atopic patient	4684	629	+	N.E.	-	-	-	1.533	This study
N4	Nasal cavity of atopic patient	4685	30	-	-	-	+	+	4.889	This study

[a] MLST profile was determined as described previously (J Clin Microbiol 38:1008-1015, 2000, https://jcm.asm.org/content/38/3/1008.long).

[b] Clonal complexes were determined using the eBURST (J Bacteriol 186:1518-1530, 2004, doi:10.1128/jb.186.5.1518-1530.2004).

[c] PCR targeted *mecA, pvl, tsst,* and *et* gene including *eta*, *etb,* and *etd* was performed by Hisata's method (J Clin Microbiol 43:3364-3372, 2005, doi:10.1128/JCM.43.7.3364-3372.2005). Six *S. aureus* strains were subjected to PCR to determine SCC *mec* typing (J Antimicrob Chemother 60:42-48, 2007, doi:10.1093/jac/dkm112).

Abbreviations: ST, Sequence typing; CC, Clonal complex; *et*, exfoliative toxin gene; *pvl*, Pantone-Valentine leucocidin gene; *tsst*, toxic shock syndrome toxin gene; N.E., not examined.

Table S2. Collection of human cutaneous bacteria used in this study

Strains	Origin	Culture media[a]	Reaction rate (ΔOD_{595}/mg/ml)	Reference
Staphylococcus aureus				
B1	Left forearm	TSA	5.356	This study
B3	Left forearm	TSA	5.844	This study
B4	Left forearm	TSA	4.6	This study
B7	Left forearm	TSA	3.022	This study
B8	Left forearm	TSA	5.156	This study
B9	Left forearm	TSA	5.044	This study
B10	Left forearm	TSA	4.6	This study
B11	Left forearm	TSA	6.444	This study
B12	Left forearm	TSA	5.8	This study
B13	Left forearm	TSA	6.311	This study
B14	Left forearm	TSA	3.511	This study
B15	Left forearm	TSA	5.733	This study
B17	Left forearm	TSA	7.778	This study
B18	Left forearm	TSA	6	This study
B19	Left forearm	TSA	3.111	This study
B20	Left forearm	TSA	4.8	This study
B21	Left forearm	TSA	3.022	This study
B22	Left forearm	TSA	3.867	This study
B24	Left forearm	TSA	2.867	This study
B30	Left forearm	TSA	1.632	This study
B31	Left forearm	TSA	8.733	This study
B40	Left forearm	TSA	0	This study

Strains	Origin	Culture media[a]	Reaction rate (ΔOD_{595}/mg/ml)	Reference
Staphylococcus epidermidis				
ATCC12228	Clinical isolation	TSA	2.685	[24]
W860371	Clinical isolation	TSA	1.156	This study
M890190	Clinical isolation	TSA	0.844	This study
A1	Forehead	BHK	0.089	This study
A2	Forehead	BHK	0	This study
A3	Forehead	BHK	2.9	This study
A6	Forehead	BHK	0.178	This study
A7	Forehead	BHK	0.933	This study
A9	Forehead	BHK	0.733	This study
A15	Forehead	BHK	0	This study
A17	Forehead	BHK	1.356	This study
A22	Forehead	BHK	0	This study
A23	Forehead	BHK	0.711	This study
A24	Forehead	BHK	0	This study
A25	Forehead	BHK	0	This study
A27	Forehead	BHK	0.822	This study
A28	Forehead	BHK	0	This study
A29	Forehead	BHK	0.489	This study
A30	Forehead	BHK	0.711	This study
A31	Forehead	BHK	0	This study
A33	Forehead	BHK	0.822	This study
C6	Dorsal skin	HTA	0.655	This study
C8	Dorsal skin	HTA	0	This study
C9	Dorsal skin	HTA	0.844	This study
C10	Dorsal skin	HTA	0.489	This study
C11	Dorsal skin	HTA	2.508	This study
C12	Dorsal skin	HTA	0.956	This study

Table S2. Collection of human cutaneous bacteria used in this study

Strains	Origin	Culture media[a]	Reaction rate (ΔOD_{595}/mg/ml)	Reference
Staphylococcus schleiferi				
B2	Left forearm	TSA	2.556	This study
B6	Left forearm	TSA	4.067	This study
B16	Left forearm	TSA	2.444	This study
Staphylococcus capitis				
A5	Forehead	BHK	0.363	This study
A12	Forehead	BHK	0	This study
A18	Forehead	BHK	5.378	This study
A19	Forehead	BHK	0.822	This study
A26	Forehead	BHK	0	This study
A32	Forehead	BHK	0	This study
A36	Forehead	BHK	0	This study
B34	Left forearm	TSA	1.644	This study
Staphylococcus hominis				
A10	Forehead	BHK	2.578	This study
A13	Forehead	BHK	0.422	This study
B26	Left forearm	TSA	3.356	This study
Streptococcus mitis				
GTC495	Clinical isolation	TSA	0.178	[29]
Pseudomonas aeruginosa				
MS5639	Clinical isolation	LB	0.089	This study
MS5640	Clinical isolation	LB	0.6	This study
MS5641	Clinical isolation	LB	0.867	This study
PAO1	Clinical isolation	LB	0.622	[30]
D4	Clinical isolation	LB	0	[30]
S10	Clinical isolation	LB	0.911	[30]
PA29	Clinical isolation	LB	0.867	[30]
Bacillus cereus				
B23	Left forearm	TSA	0.24	This study
B25	Left forearm	TSA	0.178	This study
B27	Left forearm	TSA	0	This study
B28	Left forearm	TSA	0	This study
B29	Left forearm	TSA	0	This study
B32	Left forearm	TSA	0	This study
B33	Left forearm	TSA	0	This study
B35	Left forearm	TSA	0	This study
B36	Left forearm	TSA	0	This study
B37	Left forearm	TSA	0	This study
B38	Left forearm	TSA	0	This study
B39	Left forearm	TSA	0	This study
Bacillus pumilus				
B5	Left forearm	TSA	0	This study
Bacillus subtilis				
C2	Dorsal skin	HTA	0	This study

Table S2. Collection of human cutaneous bacteria used in this study

Strains	Origin	Culture media[a]	Reaction rate (ΔOD_{595}/mg/ml)	Reference
Corynebacterium spp.				
C14	Dorsal skin	HTA	0.578	This study
C15	Dorsal skin	HTA	0.2	This study
C16	Dorsal skin	HTA	0.622	This study
C17	Dorsal skin	HTA	0.844	This study
C18	Dorsal skin	HTA	0.422	This study
C19	Dorsal skin	HTA	0.422	This study
C20	Dorsal skin	HTA	0	This study
Propionibacterium acnes				
A4	Forehead	BHK	0.044	This study
A20	Forehead	BHK	0	This study
Other *Propionibacterium* spp.				
A11	Forehead	BHK	0.244	This study
A34	Forehead	BHK	0	This study

Strains	Origin	Culture media[a]	Reaction rate (ΔOD_{595}/mg/ml)	Reference
Micrococcus spp.				
C1	Dorsal skin	HTA	0	This study
C7	Dorsal skin	HTA	0	This study
Brevibacterium spp.				
C3	Dorsal skin	HTA	0	This study
C5	Dorsal skin	HTA	0	This study

[a], Tryptic Soy Agar, TSA; Luria-Bertani media, LB; Brucella HK medium, BHK; Hoyle's tellurite agar, HTA.

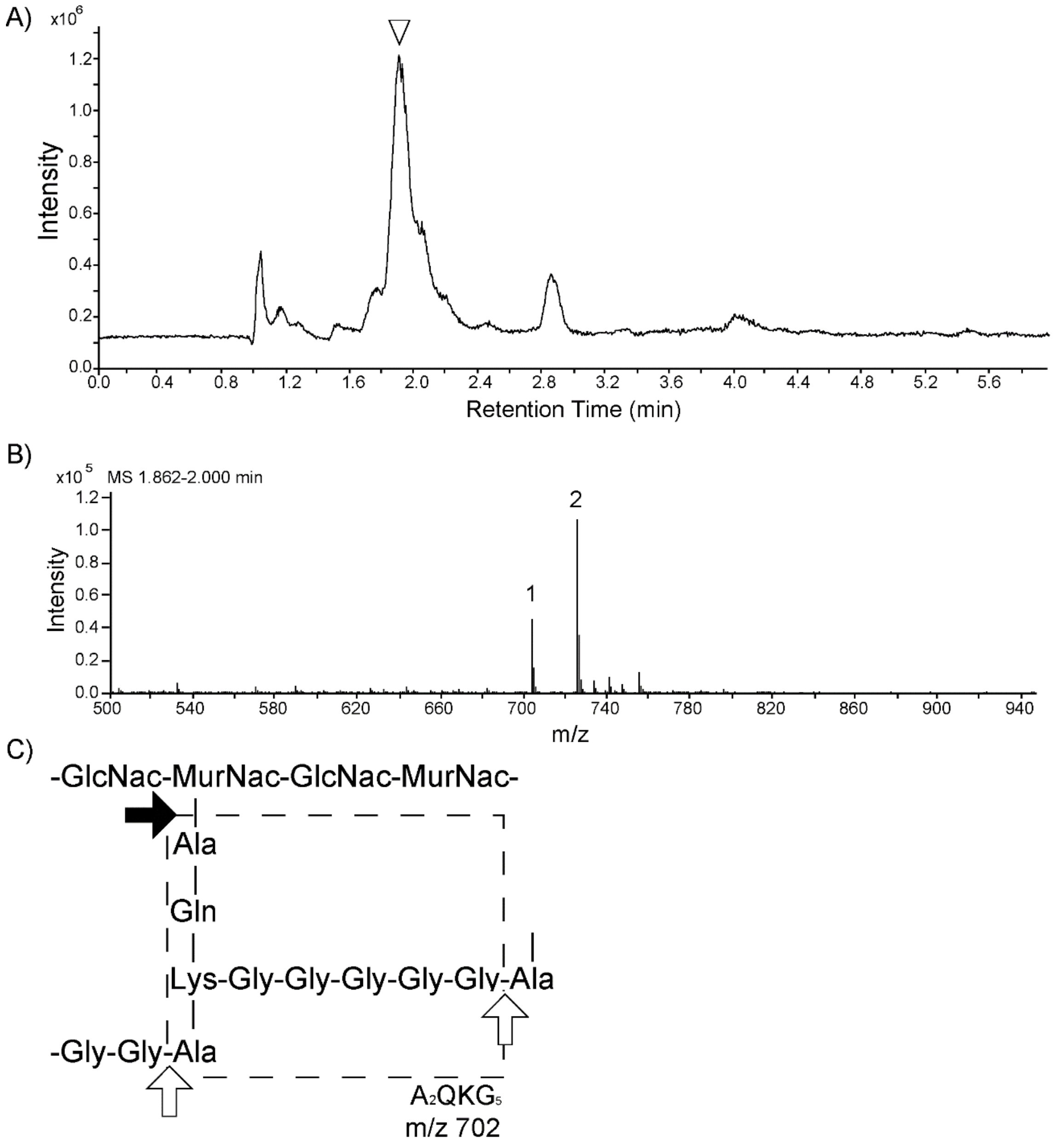

Figure S1. Analysis of *S. aureus* peptidoglycan reacted with S25-3LYS-his, using liquid chromatography coupled with quadruple time-of-flight mass spectrometry (QTOF). 2.5 mg/ml of the *S. aureus* peptidoglycan (Sigma-Aldrich, St. Louis, MO, USA) was incubated with 100 μg/ml of S25-3LYS-his at 37°C during 2 h. The digested products were separated by liquid chromatography, using an Agilent 1260 HPLC system equipped with Advance Bio Peptide Map column (1.0 × 150 mm, 2.7 μm). Two elution buffers (A, 0.1% Formic acid in water; B, 0.1% Formic acid in acetonitrile) were used with a linear gradient of buffer B (2-95%) for 10 min. After separation by liquid chromatography, the peaks were analyzed by mass spectrometry. Agilent 6530 Q-TOF LCMS System (Agilent

Technologies, Santa Clara, CA, USA) was used in this analysis. (A) Chromatograph of *S. aureus* peptidoglycan digested by S25-3LYS-his. The peak indicated by an arrowhead was subjected to mass spectrometry (MS). (B) Mass spectra of *S. aureus* peptidoglycan separated by liquid chromatography. The detected masses at peaks 1 and 2 are 702.3522 m/z and 724.3342 m/z, respectively. According to the further MS/MS analyses, peaks 1 and 2 are considered to be hydrogen and sodium adducts of A_2QKG_5, respectively. (C) Schematic diagram of S25-3LYS-his cleaved sites of *S. aureus* peptidoglycan. The linkages cleaved by S25-3LYS-his are shown by arrows. Based on the results above, the S25-3LYS-his were shown to have both an *N*-acetylmuramoyl-L-alanine amidase activity (black arrow) and a D-alanyl-glycyl endopeptidase activity (white arrow).

Turbidity change by S25-3LYS-his or control-lysate treatment between 0 min and X min is put as $\Delta OD595^{\text{S25-3LYS-his or Control solution}}_{[\text{X min}]}$.

Bacterial concentration change by S25-3LYS-his or control-solution treatment between 0 min and X min is put as $\Delta CFU/ml^{\text{S25-3LYS-his or Control solution}}_{[\text{X min}]}$.

A) Rate of turbidity reductuon (ΔOD595) $= \Delta OD595^{\text{S25-3LYS-his}}_{[\text{X min}]} - \Delta OD595^{\text{Control solution}}_{[\text{X min}]}$

B) Rate of bacterial reductuon (ΔCFU/ml) $= \Delta CFU/ml^{\text{S25-3LYS-his}}_{[\text{X min}]} - \Delta CFU/ml^{\text{Control solution}}_{[\text{X min}]}$

C) Reaction rate of S25-3LYS-his (ΔOD595/min/mg) $= \dfrac{\Delta OD595^{\text{S25-3LYS-his}}_{[\text{15 min}]}}{\text{15 (min) / Amount of S25-3LYS-his (mg)}}$

Figure S2. Mathematical formula for (A) rate of turbidity reduction, (B) rate of bactericidal reduction, and (C) reaction rate of S25-3LYS-his. The units are enclosed with round brackets.

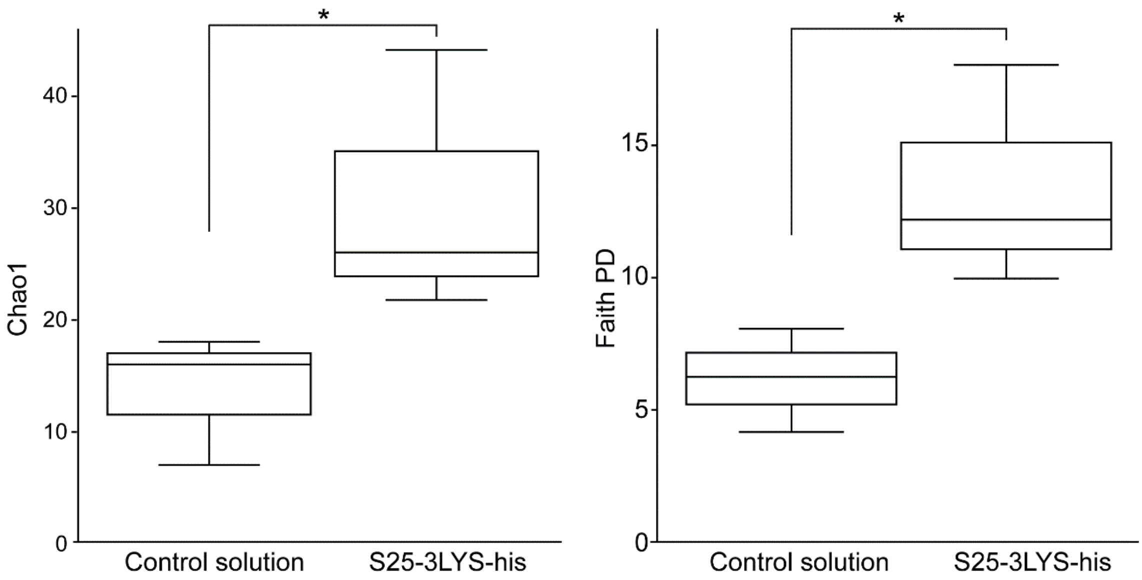

Figure S3. Alpha diversity of mouse pinna microbiome. The S25-3LYS-his-treated group was compared with control solution-treated group. On the left and right, the Chao1 and Faith-PD metrics are shown, respectively. The statistical significance is indicated as "*" ($P < 0.05$).

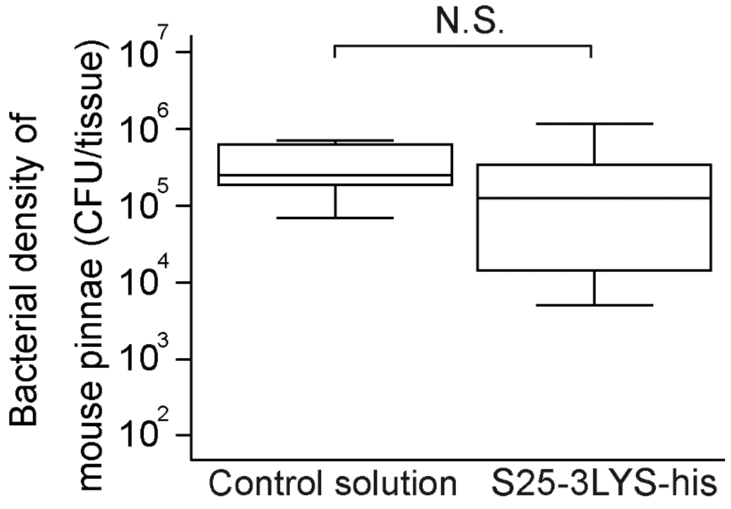

Figure S4. Bacterial density of mouse pinnae, measured by culture method. After collecting mouse pinnae in PBS, the PBS was cultured on tryptic soy agar. After the incubation (37°C, 2 days), bacterial density was measured. The control solution-treated group showed 5.43 ± 0.34 $\log_{10}$ CFU/tissue (mean ± SD; N = 3). The S25-3LYS-his-treated group showed 4.75 ± 1.11 $\log_{10}$ CFU/tissue (mean ± SD; N = 3). There was no statistical difference among these groups (Mann-Whitney U test; P, 0.0728), which is indicated by “N.S.”.
